# The study evaluating the effect of probiotic supplementation on the mental status, inflammation, and intestinal barrier in major depressive disorder patients using gluten-free or gluten-containing diet (SANGUT study): a 12-week, randomized, double-blind, and placebo-controlled clinical study protocol

**DOI:** 10.1186/s12937-019-0475-x

**Published:** 2019-08-31

**Authors:** Hanna Karakula-Juchnowicz, Joanna Rog, Dariusz Juchnowicz, Igor Łoniewski, Karolina Skonieczna-Żydecka, Paweł Krukow, Malgorzata Futyma-Jedrzejewska, Mariusz Kaczmarczyk

**Affiliations:** 1 1st Department of Psychiatry, Psychotherapy and Early Intervention, Medical University of Lublin, Głuska 1, 20-439 Lublin, Poland; 2 Department of Clinical Neuropsychiatry, Medical University of Lublin, 20-439 Lublin, Poland; 3 Department of Psychiatric Nursing, Medical University of Lublin, 20-124 Lublin, Poland; 4 Department of Biochemistry and Human Nutrition, Pomeranian Medical University, 71-460 Szczecin, Poland; 5 Sanprobi sp. z o.o. sp. k, Szczecin, Poland; 6 Department of Clinical and Molecular Biochemistry, Pomeranian Medical University, 70-111 Szczecin, Poland

**Keywords:** Depression, Probiotics, Gluten-free diet, Inflammation, Intervention study, Gut-brain axis, Gut permeability, Gut microbiota, EEG functional connectivity, Study protocol

## Abstract

**Background:**

Current treatment of major depressive disorder (MDD) often does not achieve full remission of symptoms. Therefore, new forms of treatment and/or adjunct therapy are needed. Evidence has confirmed the modulation of the gut–brain–microbiota axis as a promising approach in MDD patients. The overall purpose of the SANGUT study—a 12-week, randomized, double-blind, and placebo-controlled Study Evaluating the Effect of Probiotic Supplementation on the Mental Status, Inflammation, and Intestinal Barrier in Major Depressive Disorder Patients Using Gluten-free or Gluten-containing Diet — is to determine the effect of interventions focused on the gut-brain-microbiota axis in a group of MDD patients.

**Methods:**

A total of 120 outpatients will be equally allocated into one of four groups: (1) probiotic supplementation+gluten-free diet group (PRO-GFD), (2) placebo supplementation+ gluten-free diet group (PLA-GFD), (3) probiotic supplementation+ gluten containing diet group (PRO-GD), and (4) placebo supplementation+gluten containing diet group (PLA-GD). PRO groups will receive a mixture of psychobiotics (*Lactobacillus helveticus* R0052 and *Bifidobacterium longum* R0175), and GFD groups will follow a gluten-free diet. The intervention will last 12 weeks. The primary outcome measure is change in wellbeing, whereas the secondary outcome measures include physiological parameters.

**Discussion:**

Microbiota and its metabolites have the potential to influence CNS function. Probiotics may restore the eubiosis within the gut while a gluten-free diet, via changes in the microbiota profile and modulation of intestinal permeability, may alter the activity of microbiota-gut-brain axis previously found to be associated with the pathophysiology of depression. It is also noteworthy that microbiota being able to digest gluten may play a role in formation of peptides with different immunogenic capacities. Thus, the combination of a gluten-free diet and probiotic supplementation may inhibit the immune-inflammatory cascade in MDD course and improve both psychiatric and gut barrier-associated traits.

**Trial registration:**

NCT03877393.

## Background

Major depressive disorder (MDD) is a leading cause of disability around the world [1]. In European countries, the cost of mood disorders is estimated to be about €170 billion per year [2]. This is alarming, as more than 350 million people worldwide live with depression; worse yet, about 50% of major depressive disorder patients remain untreated [2]. A diagnosis based on the symptomatology, a lack of biological markers, failure to achieve full remission, and high percentage of suicidal patients make MDD a challenge in the twenty-first century. A high recurrence rate and a large number of non-responders justify the urgent need to look for new therapeutic approaches [3]. Traditionally considered a brain-based disease, MDD is increasingly being recognized as a systemic whole-body illness [4]. The evidence for reduction in peripheral inflammation as a promising methods for depression management is still growing [5–7].

The immune system has a powerful impact on brain function and activity [8, 9]. In 1991, Smith proposed the macrophage theory of depression, where immune and inflammatory imbalances are the main factors leading to the onset or maintenance of MDD [10]. The author suggested that the intestine is a potential immune activation gate. Bidirectional signaling via the immune, endocrine, neural, and metabolic pathways occurs between the gastrointestinal (GI) tract and brain [11]. In his earlier works, Smith termed this connection the “food-gut-allergy-behavior axis” [10]. The intestines, having one of the largest surface areas interacting with the external environment, are exposed to more antigens than any other part of the body [12]. One of the proposed causes leading to macrophage activation is an abnormal immune response to food compounds in mechanisms other than classic allergy (i.e. immunoglobulin-E (IgE)-mediated food allergy). The interplay between genetic and environmental factors may lead to the disruption of gut proteins such as tight junctions (TJs) and subsequently to the loss in epithelial wall integrity and both gut and blood-brain barrier (BBB) permeability [13]. Disruption of the intercellular TJs may be the initial phenomenon related to uncontrolled molecule translocation (i.e. toxins, antigens, and bacteria) into the blood, which promotes proinflammatory cytokine synthesis and pro/anti-inflammatory imbalance [14].

A growing amount of evidence indicates that microbiota eubiosis may be one of the potential factors supporting gut function and proper communication between the gut and the brain [15–17]. The gut microbiota performs essential functions influencing gut wall integrity (i.e. the upregulation of mucin genes, immunoglobulin A (sIgA), and cytokine secretion, suppression of intestinal inflammation, and restoration of TJs structure [18]. Gut dysbiosis affects alternations in intestinal permeability [19, 20]. Beyond the macrophage theory of depression, microbial dysbiosis is associated with impaired epithelial barrier, bacterial translocation, decreased regulatory T cells in the gut mucosa, and has been shown to promote inflammation [21]. A quantitative and qualitative recovery within the composition of microbiota could therefore have a beneficial effect on inflammation and the regulation of transport via the gut to blood circulation [22–24].

Two meta-analyses confirmed the beneficial effect of probiotics in MDD patients [25, 26]. However, the potential mechanisms of action are obscure. The proposed effect of probiotics on mental state involves not only an improvement in gut wall integrity and inflammation suppression (including the influence of bacteria-derived metabolites on the microenvironment) [27], but also regulation of the hypothalamic-pituitary-adrenal (HPA) axis, thereby affecting the response to stress modulation [28]. Neural activation of stress circuits by microbiota could directly affect central nervous system (CNS) functioning. Activation of the HPA axis and excessive cortisol secretion are potential factors leading to disruption of gut wall integrity, macrophage activation, and the secretion of proinflammatory molecules, consequently maintaining the abnormality [15, 29].

Other potential factors interacting with the gut mucosa layer are food-derived compounds, especially gluten, a complex with high immunogenic properties consisting of albumins, globulins, glutenins, and gliadins [30, 31]. Gluten proteins have been found to regulate zonulin, occludin, and claudin, all previously reported to influence intestinal permeability for macromolecules [32, 33]. Gluten is hydrolyzed by gastrointestinal proteases; however, an abundance of proline and glutamine leads to incomplete gluten degradation [34]. Gluten peptides and other food-derived compounds can be absorbed and transported into the organs by blood circulation, and could provoke an immune-inflammatory cascade [14]. In individuals with gluten-related disorders, a gluten-free diet has high potential to decrease the severity of depression symptoms [35]. However, the results of studies examining the prevalence of the abnormal response of MDD patients to food compounds are contradictory [14, 36]. Gluten restriction could lead to a significantly lower intake of whole grains [37]. Reduced consumption of cereal products may be linked with a higher risk of heart diseases. Moreover, carbohydrates derived from wheat have been shown to stimulate the activity of Bifidobacteria in the colon and their elimination could negatively impact gut bacteria specificity [38]. To sum up, a gluten-free diet is not simply either ‘pro-‘ or anti-inflammatory’ and an immune response to potential food triggers depends on individual variability [39].

The formulation with potential beneficial psychological effects is a mixture of two psychobiotic (probiotic with mental health benefits) [40]) strains: *Lactobacillus helveticus* R0052 and *Bifidobacterium longum* R0175. A study with healthy volunteers showed that supplementation with mixture of *L. helveticus* R0052 and *B. longum* R0175 decreased anxiety and depression symptoms [41]. However, no study has examined the effect of this psychobiotic mixture on both mental and somatic health in patients suffering from MDD. As a result, there is an urgent need to determine the utility and differences in the effectiveness of a gluten-free diet and probiotic supplementation, together and separately, in the management of depression.

## Methods/design

### Aim and hypothesis

The main goal of the SANGUT study (a 12-week, randomized, double-blind, and placebo-controlled study) is to determine the effect of probiotic supplementation, a gluten-free diet, and their combination on the mental state, inflammatory markers, and gut permeability markers in adult patients with MDD. The primary hypothesis is that probiotic supplementation and/or a gluten-free diet will reduce the symptoms of depression, decrease levels of inflammatory markers, and favorably affect the integrity of the intestinal mucosal barrier.

### Study design

The trial will be a prospective, randomized, double-blind (placebo = probiotic) controlled design that will last 12 weeks. The trial was registered in the clinicaltrials.gov registry (ClinicalTrials.gov identifier: NCT03877393).

### Study population

A total of 120 adult volunteers with a diagnosis of major depressive disorder (MDD) will be recruited for this study. To be eligible in the trial, subjects must fulfil all of the inclusion criteria and none of the exclusion criteria, as stated below. The following inclusion criteria will be adopted:

1. Outpatients aged 18–60 years,
2. Written informed consent to participate in this study before any study-mandated procedure,
3. Meet the DSM-5 criteria for major depressive disorder (MDD) [42],
4. Body mass index (BMI) ≥18.5 kg/m^2^ and ≤ 30 kg/m^2^,
5. MADRS (Montgomery-Asberg Depression Scale) total score of 20 points or more (moderate or severe depression) at screening (V0) and at baseline (V1), and
6. A willingness and motivation to follow the study protocol.

The exclusion criteria will be as follows:

1. Diagnosis of autoimmune, neurological, immunocompromised, thyroid, inflammatory bowel diseases, irritable bowel syndrome, diabetes, cancer, and/or IgE-dependent allergy;
2. Psychiatric comorbidities (except specific personality disorder) including mental retardation, organic brain dysfunction, or addiction (except nicotine and caffeine);
3. High risk of suicide in the investigator’s opinion;
4. An infection one month before the study baseline visit (V1);
5. The use of antibiotics and/or probiotics three months prior to the study;
6. Glucocorticosteroids and/or metformin treatment;
7. Dietary supplementation (except for vitamin D according to the “Vitamin D supplementation guidelines, 2018” [43]);
8. Changes in a pharmacotherapy and/or psychotherapy of MDD 2 weeks before the trial entry;
9. No specific (e.g. elimination, vegan, reduction) diet and changes in physical activity 4 weeks before the trial entry, and
10. Pregnancy or lactation.

Reasons for the participant to be discontinued from the study:

1. Withdrawal of informed consent,
2. Lack or incomplete compliance with the diet and/or probiotic supplementation,
3. Non-attendance at the study visits,
4. Exclusion criteria found after enrollment, and
5. Any serious adverse event during the intervention period (based on data safety monitoring).

### Sample size calculation

Based on the results of other studies evaluating probiotic strains that will be used in the present study, we assume that the mean effect of intervention will be of a medium size. As the primary outcomes will be repeatedly measured and four groups of patients will be entering the trial, there will be an approximately 81% probability of correctly rejecting the null hypothesis of no difference between the study groups with a total of 116 patients.

### Randomization

Randomization will be performed using a random sequence generator [44]. Patients will be randomized independently on antibody titers against gluten levels. The previously performed analyses will be used to check whether markers of abnormal response to gluten are useful in selecting the subgroup of patients who potentially benefit from a gluten-free diet.

Recruited individuals will be allocated into one of four groups:

1. Probiotic supplementation + gluten-free diet group (PRO-GFD; *n* = 30)
2. Placebo supplementation + gluten-free diet group (PLA-GFD; *n* = 30)
3. Probiotic supplementation + gluten-containing diet group (PRO-GD; *n* = 30)
4. Placebo supplementation + gluten-containing diet group (PLA-GD; *n* = 30)

### Dietary interventions

#### Probiotic supplementation

The probiotic groups (PRO-GFD and PRO-GD) will receive one capsule containing the probiotic mixture powder (Sanprobi Stress; Sanprobi sp. z o.o., sp.k., Szczecin, Poland) in the amount of 3 × 10^9^ colony forming units (CFU) per day divided into two equal doses. The probiotic preparation will contain two bacteria strains: *Lactobacillus helveticus* Rosell®-52, *Bifidobacterium longum* Rosell®-175 and excipients: potato starch, magnesium stearate, and the capsule shell of hydroxypropyl methylcellulose. The placebo groups (PLA-GFD and PLA-GD) will receive the same capsule containing only the excipients, i.e. maize starch, maltodextrins, and the capsule shell. The placebo will be indistinguishable in color, smell, and taste from the probiotic formulation. Participants will be asked to consume the supplements before breakfast. Each of the study participants will be asked to collect the blister packs to evaluate compliance after the study.

#### Elimination diet

The patients in the GFD groups will follow the elimination diet containing no gluten. During the Baseline Visit (V1), participants will be educated about the rules of gluten-free diet restrictions and informed that the elimination diet is not a caloric restriction diet so the consumption of nutrients should not be changed. Information on the origins of gluten, the grains containing gluten, and highly processed food with gluten, will be received by all participants. They will be encouraged to consume natural, fresh foods and read the labels of food products in order to evaluate the gluten content. To avoid potential outcome bias resulting from a balanced diet and change in dietary habits, participants will be asked to follow their usual eating patterns, excluding gluten-containing products.

Before the intervention is implemented (between V0 and V1), patients will be asked to keep a diet diary (a 3-day food record). Changes and modifications in typical food consumption (based on the 3-day food record) during the consultation (at baseline, V1) will be proposed by a dietitian. Moreover, each participant will receive a list of products to be eliminated as well as a list of substitutes with the same taste, organoleptic properties, and nutritional values. A guide on the gluten-free diet and seven proposed menus for breakfast, lunch, dinner, and snacks will be given to each participant. The diet protocol will include only qualitative data, without the portion sizes, to keep the amount of food consumed at the current level. Patients will be educated about sticking to a diet by a dietitian during a consultation (visit V1).

### Data collection and methods

#### Tools

The following clinical/dietary information will be obtained:

1. Socio-demographic Data (a self-prepared questionnaire)
2. Dietary habits: Food Frequency Questionnaire (FFQ-6) [45], a 3-day food record
3. Physical activity: The International Physical Activity Questionnaire (IPAQ) [46]
4. Smoking status by breath carbon monoxide levels in exhaled air
5. Vital signs: Heart rate (HR), blood pressure (RR), and core body temperature
6. Anthropometric measures: Weight, height, body mass index (BMI), waist-to-hip ratio, and body composition
7. The severity of depressive symptoms: Montgomery-Asberg Depression Scale (MADRS) [47], and Beck’s Depression Inventory (BDI) [48]
8. Psychological problems and symptoms of psychopathology: Symptom Checklist-90 Questionnaire (SCL-90) [49]
9. Quality of life: Short Form (36) Health Survey (SF-36) [50]
10. Gastrointestinal symptoms: Gastrointestinal Symptom Rating Scale (GSRS) [51]
11. Subjective assessment of stress levels: Perceived Stress Scale (PSS-10) [52] and Childhood Trauma Questionnaire (CTQ) [53]

In the blood serum, we will evaluate markers of:

1. Gut barrier integrity: Intestinal fatty acid-binding protein (IFABP/FABP2), and lipopolysaccharide binding protein (LBP)
2. Gluten sensitivity: Immunoglobulin G and immunoglobulin A anti-gliadin antibodies (anti-AGA IgG/IgA), and IgG anti-tissue transglutaminase (anti-tTG2)
3. Inflammation: High-sensitivity C reactive protein (hs-CRP), tumor necrosis factor alpha (TNF-alpha), and interleukins IL-6 and IL-1 beta
4. HPA axis activity: Cortisol
5. Metabolic indices: Total cholesterol, low density lipoprotein (LDL) cholesterol, high density lipoprotein (HDL) cholesterol, triglycerides (TG), glucose, and insulin
6. Liver function: Alanine aminotransferase (ALT) and asparagine aminotransferase (AST)
7. Excluded diseases: Hematopoietic system (peripheral blood morphology), thyroid diseases (thyroid stimulated hormone, TSH), diabetes (hemoglobin glycosylated, HbA1c), coeliac disease (total-IgA and IgA antibodies against tissue transglutaminase, anti-TG2), and allergy (total-IgE antibodies levels).

The following analyses will be conducted in the stool: gut microbiota (taxonomic and functional analysis) and concentrations of short-chain fatty acids (SCFAs).

Activity of the brain and its relationships with implemented interventions will be assessed with resting-state electroencephalography (EEG). After collecting raw EEG recordings, they will undergo mathematical analysis to obtain the quantitative parameters characterizing neural activity which can be evaluated with reference to applied experimental effects.

### Methods of assessment

#### Dietary assessment and diet adherence

Diet assessment and adherence to the protocol will be examined using the Food Frequency Questionnaire with six answers (FFQ-6) validated on a Polish population [45]. The FFQ questionnaire will be used to assess intake of 62 products (including the main gluten sources) divided into eight groups that are consumed in Poland. Respondents will have to select one out of six frequency categories: (1) never or almost never, (2) once a month or more rarely, (3) several times a month, (4) several times a week, (5) daily, and (6) several times a day. The FFQ questionnaire will be filled out by a clinical dietitian with experience in conducting nutritional interviews. Using the “Album of photographs of food products and dishes” [54], participants will determine the serving sizes of the consumed products. The amount of food intake which is not included in the aforementioned album will be determined using the ilewazy.pl website [55]. Patients will also be asked to complete a 3-day food record (including two weekdays and one weekend day) before the Baseline Visit (V1), and twice in the study period (first between 1 and 6 weeks from the implementation of the diet, and for the second time between 6 and 12 weeks from the implementation). Based on the food records, we will be able to estimate the nutritional value and assess the intake and percentage of consumed nutrients from the recommended amounts. According to the method from earlier studies (multiplying protein contained in gluten cereal by 0.8) [56, 57], gluten in patients’ diet will be estimated from the FFQ questionnaire and 3-day food records. Based on the FFQ results, alcohol consumption will also be estimated. A possible influence of other nutritional factors (energy value, nutrient intake) on the obtained results will be checked before and during the intervention based on dietary diaries.

#### Other lifestyle factors

Physical activity will be assessed using the International Physical Activity Questionnaire (IPAQ) which allows for expressing physical activity in the metabolic equivalent – MET-min/week [46]. The IPAQ estimates physical activity of different intensity levels (walking, moderate-intensity, and vigorous-intensity activities) during the last seven days. Based on the results, the responders are classified into one of three activity categories: ‘insufficient,’ ‘sufficient,’ or ‘high’. The level of physical activity is a potential confounder having an impact on gut microbiota composition [58, 59] and some biochemical parameters [60, 61]. Studies have also shown that excessive workouts affect gut permeability [59].

Smoking status will be evaluated by means of the Micro+Smokerlyzer (Bedfont Scientific Ltd., Station Road, Harrietsham, Maidstone, Kent, ME17 1JA, England) which is a quick method to analyze carboxyhemoglobin and carbon monoxide parts per million (ppm) levels in exhaled air. The monitor uses an electrochemical sensor, has a concentration range of 0 to 500 ppm, repeatability of less than ±5%, and an accuracy of 2 ppm. Evidence indicates that smoking dysregulates gut microbiome and leads to low-grade inflammation [62, 63].

#### Vital signs

Systolic and diastolic blood pressure and pulse rate will be measured using the standard digital meter in a seated position, after at least five minutes of rest, three times (in 1-min intervals) by placing the cuff on the left upper arm. The first measurement will be discarded. During the same visit, vital sings will be taken prior to blood sampling. Core body temperature will be monitored using a forehead digital thermometer. Abnormalities of clinical significance will be recorded as an adverse event.

#### Anthropometric measurement

The measurement of waist and hip circumferences (repeated twice at each visit) will be performed according to the World Health Organization (WHO) protocol [64]. Weight, body mass index (BMI), fat mass, muscle mass, and total body water will be measured using a segmental multifrequency bioimpedance analyzer (Tanita BC-601; Tanita Corp., Tokyo, Japan). All anthropometric data will be recorded after an overnight fast. Patients will be weighed while wearing light clothing and without metal objects (i.e. belt, jewelry). Participants will be asked to wear a similar amount of clothes on each visit. Evidence confirms a link between anthropometric parameters and other parameters assessed in our study [65–67]. Changes in anthropometric data will be considered as potential factors linked with metabolic parameters, inflammatory and gut permeability markers, gut microbiota composition, and physical and mental state. There is some weak evidence that gut microbiota may have an impact on body weight [68].

#### Patients’ well-being

To check the effect of the intervention on the severity of depression, other psychiatric features, co-morbid symptoms, and general patient health, the following scales and questionnaires will be used:

1. MADRS scale (Montgomery-Asberg Depression Rating Scale) [47] for clinical assessment of depression severity will be administered by a well-trained psychiatrist. The MADRS is a 10-item scale that includes questions concerning apparent sadness, reported sadness, inner tension, reduced sleep, reduced appetite, concentration difficulties, lassitude, inability to feel, pessimistic thoughts, and suicidal thoughts. The assessment will always be performed by the same person blinded to the intervention type.
2. BDI (Beck’s Depression Inventory [48]) for self-reporting measurement of the severity of depression. The BDI consists of 21 multiple-choice questions.
3. SCL-90 (The Symptom Checklist-90 Questionnaire [49]) assesses the general intensity of psychopathological impairment. The SCL-90 is a self-reporting measurement tool that includes 90 items regarding the currently felt symptoms divided into nine subscales: somatization, obsessive-compulsive, interpersonal sensitivity, depression, anxiety, hostility, phobic anxiety, paranoid ideation, and psychoticism.
4. PSS-10 (The Perceived Stress Scale [52]) includes 10 items and measures the self-reported perception of psychological stress in respondents by asking about their thoughts and feelings during the last month.
5. CTQ Questionnaire (Childhood Trauma Questionnaire [53]) is used for the self-assessment of traumatic experiences which covers 28 items in total and consists of five subscales addressing: physical, emotional, and sexual abuse, and physical and emotional neglect.
6. SF-36 Health Survey (The Short Form (36) Health Survey [50]) includes patients’ reports about their vitality, physical functioning, bodily pain, general health perceptions, physical role functioning, emotional role functioning, social role functioning, and mental health.
7. The GSRS scale (The Gastrointestinal Symptoms Rating Scale [51]) is a self-administered questionnaire which assesses five symptom clusters (combined from 15 items) depicting: reflux, abdominal pain, indigestion, diarrhoea, and constipation experienced over the past week.

#### EEG

Electroencephalography (EEG) is a non-invasive method enabling the assessment of electrophysiological neural activity. Subjects participating in the study will sit with closed eyes throughout the EEG recording. The assessment will be carried out during the daytime, between 10:00 a.m. and 2:00 p.m. to ensure that participants will remain awake. The EEG recordings, after the initial calibration of the equipment, will last about 15 min so that after cleaning, we will have at least five minutes of pure electrophysiological recording available from each participant. To ensure study comparability and reproducibility of the study, a standard procedure will be conducted, with 19 electrodes placed according to the International 10–20 system [69, 70].

Oscillatory activity of the central nervous system may be analyzed in order to extract relevant information regarding the functioning of the brain. Resting-state EEG assesses spontaneous brain activity organized as a synchronized, or in the case of brain pathology, a desynchronized, system. The evaluation of task-free neural behavior allows a qualitative, clinical assessment of parameters such as the dominant frequency, the occurrence of pathological phenomena such as seizures, or abnormal brain response to stimulation. Digitized EEG recordings can be analyzed with advanced mathematical methods to assess the functional connectivity and organization of intrinsic neural networks. Functional connectivity (FC) refers to the process of neural synchronization that functionally integrates the cortical areas necessary to maintain consistent and effective brain activity. Based on various properties of the EEG signal, e.g. phase coupling, by applying non-linear mathematical methods (e.g. phase lag index, PLI) [71], it is possible to assess changes in the synchronization patterns as an effect of a given experimental intervention as well as those that reduce the inflammatory states within an organism. The most advanced analyses of the EEG signal concern the modelling of brain activity in the form of functional neural networks [72]. Network analysis enables the reconstruction of synchronized neural behavior as a global system of inter- and intra-cortical interactions, and not only as a given set of connections. Due to the implementation of the network approach, it is also possible to distinguish cortical areas, so-called hubs, that play a key role in neural integration. Their location and strength are critically important indicators of how the brain functions as an information processing system. Considering the above, we will apply the PLI as a functional connectivity measure and the network indicators adopted from graph theory such as centrality, network assortativity, metrics indicative of path length, and small-worldness [73].

Changes in FC have also been studied in patients with MDD [74, 75] and have shown that these EEG markers may have potential value as biomarkers. This concept created a new clinical possibility of considering MDD symptoms in connection with gut microbiota and structural and functional changes in the brain.

### Sampling

#### Blood collection

Venous blood will be collected by qualified nurses. The collection will be performed under conditions of fasting, upon overnight resting, in the morning, between 8:00 and 10:00 a.m., to minimize the effect of changes in daily cortisol production. To obtain serum, blood will be centrifuged at 1500×*g* for 10 min. Until analysis, the blood samples will be preserved in 2 mL Eppendorf tubes and frozen at a temperature of − 80 °C.

#### Stool collection

Participants will be asked to collect stool samples after overnight fasting to avoid the effect of food consumption on short-chain fatty acid synthesis. Each participant will receive detailed instructions describing the method for material collection. A plastic holder will be used to collect faeces into a sterilized screw-capped collection container. Participants will be asked to store the sample in a fridge and deliver the sample up to 24 h after collection to the clinic. The samples will be preserved in 2 mL Eppendorf tubes containing 600 μL DNA/RNA later solution (Zymo Research, Freiburg, Germany) to prevent the degradation processes and will be frozen at − 80 °C until the beginning of the microbiota analyses.

DNA extracted from the stool will be amplified using primers flanking the hypervariable region of the 16S rRNA gene. 16S amplicons will be sequenced using an Illumina MiSeq platform (Illumina Inc., San Diego, CA, USA) and the raw reads will be processed using the Mothur pipeline (version 1.39.5) [76]. The SILVA 132 rRNA database [77] will be used to assign taxonomy. To predict a microbial community metagenome and its functional potential based on the 16S data, the PICRUSt software package will be used [78].

**Fig. 1:**
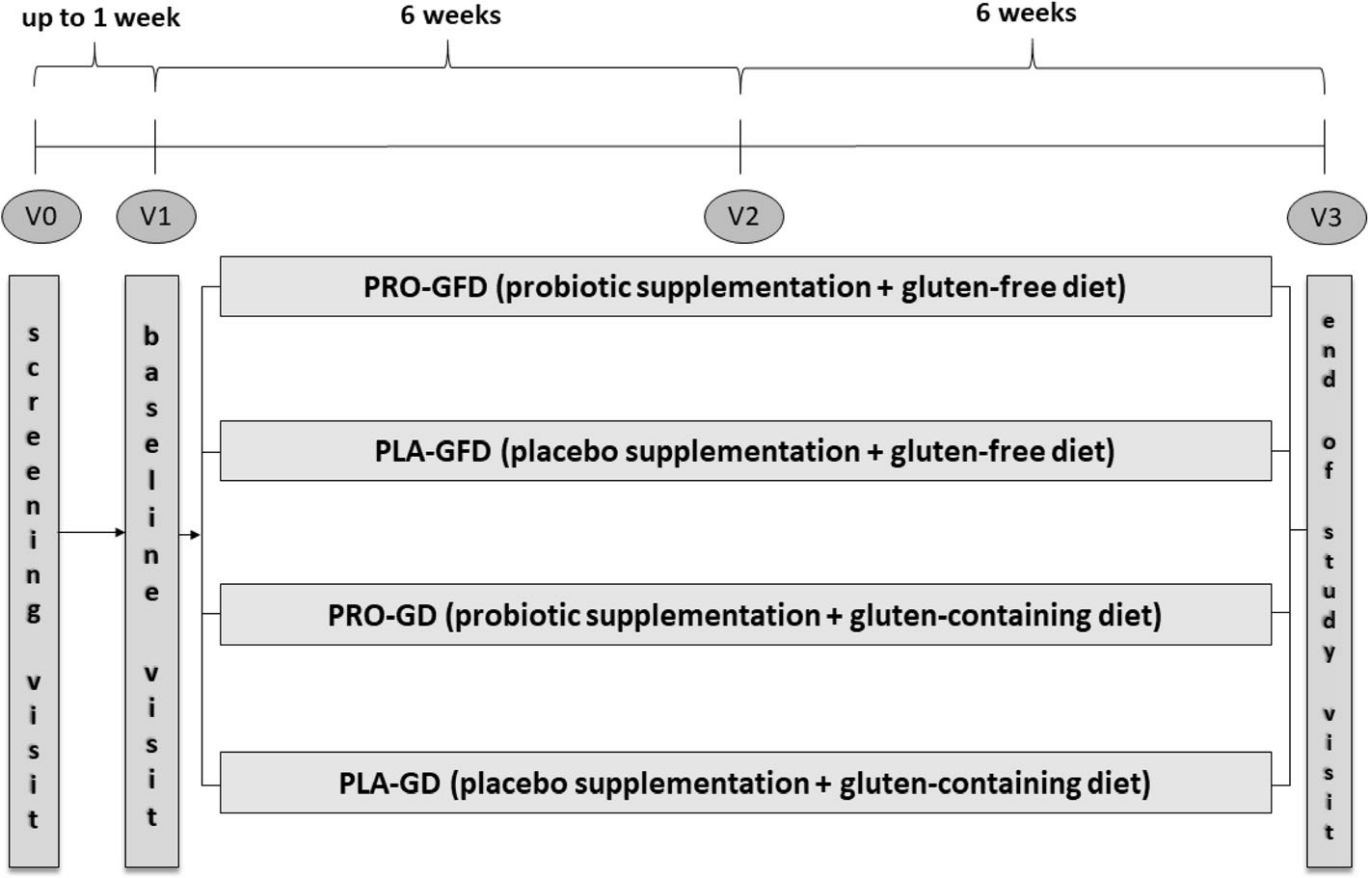
Timeline of SANGUT STUDY visits. V0 – Screening visit; V1 – Baseline Visit; V2 – Visit 2; V3 – End of Study Visit

An overview of the study design and assessments to be conducted during the study and their timing is presented in Fig. 1 (study design) and Table 1 (assessment point times and examined variables).

**Table 1:** Schedule of study procedures

	Screening	Randomization	Intervention Period	End of the study
Visit Number	V0	V1	V2	V3
Visit time and Window	Day –7 to –1	Day 0	6 weeks ±3 days	12 weeks ±3 days
Informed consent	+			
Inclusion/exclusion criteria	+	+		
Somatic examination	+	+		
Neurological examination	+	+		
Socio-demographic characteristics	+			
Medical history	+			
Psychiatric history	+			
Depression symptoms severity	+	+	+	+
Stress level	+	+	+	+
Quality of life	+	+	+	+
Gastrointestinal symptoms	+	+	+	+
Dietary assessment	+	+	+	+
Physical activity	+	+	+	+
Anthropometric measures	+	+	+	+
Vital measures	+	+	+	+
Smoking status	+	+	+	+
Blood sample	+			+
Stool sample	+			+
Electroencephalography	+			+
Adverse events report		+	+	+

### Data management and analysis plan

#### Data analysis principles

To evaluate the efficacy of the intervention on the well-being of patients, the following changes from the baseline (V1) to the end of the study (V3) will be determined as primary outcomes:

1. The severity of depression symptoms:
  - MADRS total score
  - BDI total score
2. The severity of psychopathological impairment:
  - SCL-90 total score
3. The quality of life:
  - SF-36 total score
4. Stress levels:
  - PSS-10 total score

To evaluate the effect of the intervention on physiological parameters [79], the following changes will be determined as secondary outcomes:

1. In serum, from the screening (V0) to the end of the study (V3), levels of:
  - proinflammatory biomarkers: hs-CRP, Il-6, Il-1beta, and TNF-alpha
  - gluten sensitivity biomarkers: anti-TG2 IgG antibodies and anti-AGA IgG/IgA antibodies
  - intestinal permeability biomarkers: I-FABP/FABP-2 and LBP
  - metabolism biomarkers: total cholesterol, LDL cholesterol, HDL cholesterol, TG, glucose, and insulin
  - HPA axis biomarker: cortisol
  - liver function biomarkers: ALT and AST
2. In stool, from the baseline (V1) to the end of the study (V3):
  - taxonomic and functional analysis of gut bacterial community
  - SCFAs levels
3. Brain activity, from the baseline (V1) to the end of the study (V3):
  - EEG analysis
4. Gastrointestinal symptoms, from the baseline (V1) to the end of the study (V3):
  - GSRS scale.

### Monitoring data collection

All study team members will be trained by qualified, well-experienced professionals in areas referring to the planned measurements for the accurate performance of the procedures, data collection, and adherence to the study. Previously established procedures for data collection will be used (see Methods of Assessment section). The quality and correctness of the data collection will be checked by a researcher completing a structured online spreadsheet after each visit. Assessment of the electronic document will be performed once a week by a supervisor.

### Statistical analysis

Statistical analysis will be performed according to the E9 Statistical Principles for Clinical Trials [80]. For quantitative outcomes, the between-group comparisons will be performed using the ANOVA or Kruskal-Wallis rank-based nonparametric test depending on the data distribution followed by appropriate post-hoc tests. The qualitative outcomes will be compared using the Chi-square test. Longitudinal data will be analyzed with either repeated measures of ANOVA or aligned rank-transform ANOVA, depending on the data distribution, or McNemar’s test.

The analysis of the gut microbial community will include alpha diversity, beta diversity, ordination techniques (a principal coordinate analysis), and taxonomic and functional differences. Alpha diversity will be measured by means of the Chao1 (richness) and Shannon (richness and evenness) indices. All indices will be determined using originally observed count data (without data pre-processing) and comparison within the groups (V0–V3) and between the groups at the end of the study (at V3) using the non-parametric Wilcoxon signed-rank test or Mann-Whitney (or Kruskal-Wallis) test, respectively. Beta-diversity will be measured by means of the Bray-Curtis dissimilarity metric and the weighted UniFrac distance metric. Beta-diversity analysis will be preceded by the removal of rare species and normalization to account for unequal library sizes. The between-group differences in gut microbial composition will be visualized using principal coordinate analysis based on the Bray-Curtis dissimilarity and UniFrac distances. The permutational multivariate analysis of variance (PERMANOVA) will be conducted on the Bray-Curtis and weighted UniFrac dissimilarity matrices to assess the group-level (at V3) differences. Taxonomic differential abundance will be tested by comparing the fractional abundances. The analysis of alpha and beta diversity, ordination analysis, and taxonomic differential abundance will be conducted using the R (version 3.5.1) package Phyloseq [81]. The PERMANOVA+ add-on package of PRIMER 7 (version 7.0.13) will be used as analysis tools to compare beta diversity. To predict the metagenome functional content using the output of the 16S rRNA analysis pipeline, the software package PICRUSt will be used. Functional abundance differences will be analyzed and visualized using STAMP [82]. To account for multiple testing, FDR-adjusted *p* values (q values) will be reported.

### Project Management

#### Timeline of the study

This study will consist of four visits (see Table 1 and the Methods of Assessment section for a detailed description of the framing and all procedures performed during each visit):

1. V0: Screening Visit to screen and enroll eligible patients into the study;
2. V1: Baseline Visit up to 1 week after V0 to randomize patients to one of four arms of the study;
3. V2: Visit 2, during the intervention period, after six weeks (±3 days) from Baseline Visit;
4. V3: End of Study Visit to complete all procedures in this study, after 12 weeks (±3 days) from Baseline Visit.

The total duration from the screening to the end of the study will be 13 weeks at maximum.

#### Data safety monitoring

Based on anthropometric measurements, vital signs, and self-reported patient symptoms recorded at the visits during the intervention period (V2 and V3), the long-term safety and tolerability of probiotics and gluten elimination will be assessed. Participants will be asked to document any suspected adverse events in written form and to report them at the next visit.

## Discussion

A potential connection between abnormal immune response, food-derived compounds, and MDD is still poorly understood; however, increasing evidence has confirmed the importance of the interplay between microbiota, gut permeability, immune-inflammatory processes in the pathophysiology of MDD [83]. The modulation of the gut-microbiota-brain axis could therefore be a promising therapeutic target for mental illnesses [15, 16, 84]. This concept has produced a new clinical approach involving dietary interventions restoring gut eubiosis in the treatment of MDD.

A gluten-free diet and probiotic supplementation, separately and in combination, have received much attention as a potential therapeutic strategy. This approach is of particular interest as it considers that a gluten-free diet changes the microbiota profile and alters the activity of microbiota-mediated biochemical pathways [85, 86]. Notably, the microbiota is able to digest gluten. Bacterial and fungi enzymes are involved in gluten break-down and the formation of peptides with different immunogenic capacities [87, 88]. Hence, gut dysbiosis could intensify the immunogenic effect of cereal-derived compounds [89]. Studies have confirmed differences in gut microbiota of patients with celiac disease and non-celiac gluten sensitivity (NCGS) [90, 91]. Eubiosis is necessary for both appropriate gut function and restoration of intestinal barrier integrity, [87, 88, 92].

This bidirectional interaction between a GFD and microbiota suggests that the combination of a gluten-free diet and probiotic supplementation is essential for the inhibition of the immune-inflammatory cascade. However, whether the separate use of one of these interventions or their combination will be the most effective treatment strategy to regulate CNS and digestive tract functions in MDD patients has yet to be determined. Results confirming the efficacy of such a therapeutic approach would provide support for the introduction of dietary interventions as an integral part of treatment in psychiatric wards.

To the best of our knowledge, this will be the first intervention study assessing the efficacy of both probiotic supplementation and a gluten-free diet and comparing the effect of both interventions and their combination in MDD patients. The protocol and the outline were developed by a multidisciplinary researcher team. The methodology employed in the study benefited greatly from our diverse set of knowledge, experience and perspectives. Considering the highly rigoristic selection criteria, the participant group is relatively large. Rigorous procedures established for this trial will be followed by well-trained researchers to ensure that research will be conducted in accordance with standards of best practice. The measurements listed above will be introduced to reduce the potential bias related to confounding variables (e.g. smoking, physical activity, and diet).

There may be some possible limitations in the design of the study:

First, the impossibility of blinding the diet is a factor that could affect assessment of both the participant and investigator. For this reason, the researcher evaluating the severity of symptoms will not be informed as to whether the patient will be allocated to the GFD or GD arm of intervention.

Second, the lack of motivation is a symptom commonly experienced by MDD patients and could contribute to the non-attendance at the study visits, incomplete implementation of the intervention, or failure to complete the intervention.

Third, patients in the GFD groups receiving dietary advice might try to introduce other changes in food consumption (e.g. reduce the food portion size and/or consume more vegetables and fruits). Therefore, health improvement may not be specific to the implemented gluten-free diet.

Finally, recall bias in self-reporting is another potential confounder of the study. However, dietary consultation and food intake assessment during the study period should minimize the likelihood of its occurrence.

## Data Availability

The datasets used and analyzed during the study will be available from the corresponding author on request.

## Abbreviations

AGA: Anti-gliadin antibodies
ALT: Alanine aminotransferase
anti TG2: antibodies against tissue transglutaminase
AST: Asparagine aminotransferase
BBB: Blood brain barrier
BDI: Beck’s Depression Inventory
BMI: Body mass index
CNS: Central nervous system
CTQ: Childhood Trauma Questionnaire
DSM-5: Diagnostic and Statistical Manual of Mental Disorders, fifth edition
EEG: Electroencephalography,
FC: Functional connectivity
FFQ: Food Frequency Questionnaire
GD: Gluten containing diet
GFD: Gluten-free diet
GI: Gastrointestinal
GSRS: Gastrointestinal Symptom Rating Scale
HbA1c: Hemoglobin glycosylated
HDL: High density lipoprotein
HPA: Hypothalamic-pituitary-adrenal
HR: Heart rate
hsCRP: high-sensitivity C reactive protein
IFABP: Intestinal fatty acid-binding protein
IgE: Immunoglobulin-E
IPAQ: International Physical Activity Questionnaire
LBP: Lipopolysaccharide binding protein
LDL: Low density lipoprotein
MADRS: Montgomery-Asberg Depression Scale
MDD: Major depressive disorder
PLA: Placebo
PPM: Parts per milllion
PRO: Probiotic
PSS-10: Perceived Stress Scale
RR: Blood pressure
SCFAs: Short chain fatty acids
SCL-90: Symptom Checklist-90 Questionnaire
SF-36: Short Form (36) Health Survey
sIgA: secretory immunoglobulin A
TG: Triglycerides
TJs: Tight junctions
TNFα: Tumor necrosis factor alpha
TSH: Thyroid stimulated hormone
V1: Baseline Visit
V1: Consultation Visit
WHO: World Health Organization
